# Association between Body Mass Index, Waist-to-Height Ratio and Adiposity in Children: A Systematic Review and Meta-Analysis

**DOI:** 10.3390/nu8080512

**Published:** 2016-08-20

**Authors:** Nerea Martin-Calvo, Laura Moreno-Galarraga, Miguel Angel Martinez-Gonzalez

**Affiliations:** 1 Department of Preventive Medicine and Public Health, University of Navarre, Pamplona 31009, Spain; nmartincalvo@unav.es; 2 Navarra Institute for Health Research (IdisNa), Pamplona 31008, Spain; lauramoreno11@yahoo.es; 3 CIBER Physiopathology of Obesity and Nutrition (CIBERobn), Carlos III Institute of Health, Madrid 28029, Spain; 4 Department of Pediatrics, Complejo Hospital de Navarra, Pamplona 31008, Spain

**Keywords:** body mass index, waist-to-height ratio, DEXA, body fat, children, obesity

## Abstract

Obesity is defined as an abnormal or excessive fat accumulation that may impair health. Dual-energy X-ray absorptiometry (DEXA) has been suggested as the gold standard to define obesity, but because its use is complex and expensive, anthropometric measures such as body mass index (BMI) or the waist-to-height ratio (WtHr) have been used as alternatives. The aim of this study was to review the published literature and investigate the correlation of BMI and WtHr with body fat (BF) measured by DEXA in pediatric populations. References were sought in PubMed/Medline and Embase datasets. Five original articles, published between 2013 and 2015, were finally included in this review. Their sample size ranged from 83 to 5355, and the age of participants ranged from 4.9 to 19 years old. The most frequently reported association measurements were the coefficients of determination (*R*^2^), followed by correlation coefficients and least-squares regression coefficients. BF measured by DEXA was strongly correlated with both BMI (*R*^2^ ranging from 0.32 to 0.91) and WtHr (*R*^2^ ranging from 0.49 to 0.73). Thus, either BMI or WtHr may be useful to define obesity when more sophisticated techniques are not available. Our systematic review of the available literature found that neither index demonstrated superiority in assessing obesity in children.

## 1. Introduction

Overweight and obesity are defined as abnormal or excessive fat accumulation that may impair health [1]. Worldwide obesity has more than doubled since 1980, and in 2013, 42 million children under the age of five were overweight or obese [1]. Obesity has been related to several chronic diseases in adulthood such as cardiovascular disease, type 2 diabetes mellitus (T2DM) and metabolic syndrome. Obese children are more likely to become obese adults [2], and the biological changes that lead to obesity-related cardio-metabolic disease start to develop in childhood [3,4,5,6,7]. In addition, hypertension, left ventricle hypertrophy, and high serum lipids have already been described in children with obesity [4,5,6,7,8]. Other obesity related disorders such as T2DM, depression, sleep disorders, and asthma have been observed in children as well [3,4,6,7,8,9].

Body composition can be assessed by techniques such as air displacement plethysmography, bioelectrical impedance analysis, and dual-energy X-ray absorptiometry (DEXA). While DEXA is frequently used as the reference standard to assess body composition in children [10], and it has been suggested as the gold standard [11], its complexity and cost limit its use in daily clinical practice and epidemiological research.

On the other hand, anthropometric measures such as body mass index (BMI), waist circumference (WC), and waist-to-height ratio (WtHr) can be easily-obtained and can serve as inexpensive tools to identify general obesity [12]. BMI has been largely used for the definition of obesity in children because it correlates well with body fat (BF) [13] and cardiovascular risk factors [14,15]. This index, however, has some notable limitations: it does not differentiate between fat mass and fat-free mass [16], it does not take into account fat distribution, and its correlation with BF depends on nutritional status, being stronger in overweight and obese children compared to that in lean ones [17]. Several studies have suggested that WC and WtHr may be convenient to assess obesity because they correlate better than other indices with visceral abdominal fat [18], which is considered as the most dyslipidemic and most atherogenic manifestation of adiposity [19], and with metabolic risk factors in children [20,21].

Whether WtHr is superior to BMI for the assessment of general obesity is still unclear. The aim of this study was to review studies that assessed the correlation of both BMI and WtHr with BF measured by DEXA in pediatric populations.

## 2. Materials and Methods

The studies in this systematic review where chosen according to the protocol described in [22] that defines the search strategy, the inclusion criteria, the information extraction methods, and the statistical analyses used in our quantitative synthesis of the literature.

**Figure 1:**
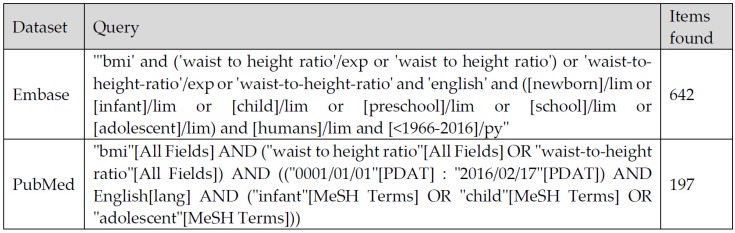
Search strategy based on Key words for PubMed/Medline and Embase databases.

References were sought in PubMed/Medline and Embase datasets using specific keywords related to the topic of interest. The parameters of the search included the following filters: language (English) and age (up to 18 years old). The search was performed in February 2016. No time period limit was established. The strategy used in this search is shown in Figure 1.

**Figure 2:**
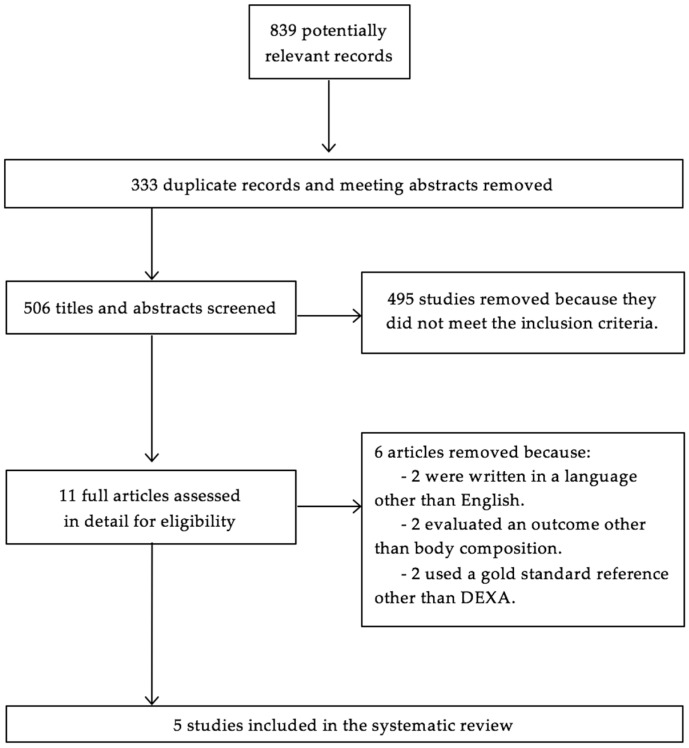
Flow diagram of the relevant studies selection process.

We identified 839 potentially relevant records. After removing duplicates and excluding abstracts to conferences, we obtained a list of 506 articles. Martin-Calvo N., and Moreno-Galarraga L. independently screened titles and abstracts and decided upon the eligibility of studies based on the features of interest. In order to be included, the article must have reported data about the measurements of both BMI and WtHr, and must have used DEXA as the reference standard. We excluded reviews, editorials, letters, and meeting abstracts. We also excluded studies reporting only one of the anthropometric measures of interest, studies that did not use DEXA as the reference method, and studies reporting only data for trunk fat or abdominal fat. Discrepancies were solved by consensus and by asking Martinez-Gonzalez M.A. Finally, Martin-Calvo N. assessed in detail 11 full-text articles for eligibility. After following the selection strategy shown in Figure 2, we selected five articles in total.

We followed the PRISMA (Preferred Reporting Items for Systematic Reviews and Meta-Analyses) guidelines to conduct this review [23]. The methodological quality and risk of bias were assessed by reading the articles and using QUADAS (Quality Assessment of Diagnostic Accuracy Studies), a tool for quality assessment of studies of diagnostic accuracy included in systematic reviews [24]. The tool is structured as a list of 14 questions such as “were selection criteria clearly described?” and “is the reference standard likely to correctly classify the target condition?” Each question had to be answered “yes”, “no”, or “unclear”. The QUADAS tool is simple and quick to complete, and does not incorporate a score.

We extracted from the five identified articles the data concerning the purpose of the study: authors, study design, sample size, sample characteristics (age, sex, and ethnicity-race), sample mean body fat percentage (%BF), variable used to report the BF, and statistical results (*R*^2^, coefficients of regression and the area under the receiver operating characteristic (ROC) curve). When studies reported several models with different degrees of adjustment, we extracted all of them in order to get further information. Analyses that exceeded or did not fit the purpose of this review were not considered.

When the correlation coefficient (*r*), but not the *R*^2^, was given, the latter was calculated as the square of the *r* [25]. We also calculated the 95% confidence interval for the *R*^2^ in all the studies using Fisher’s *z*’ transformation of the correlation coefficient (*z*’ = 0.5[ln(1 + *r*) − ln(1 − *r*)]). We performed a quantitative meta-analysis to obtain a pooled *R*^2^ for each anthropometric measure with DEXA, and we compared those pooled estimates using as standard error for the comparison of both *z*’ and the standard error for the comparison of two *z* according to the Fisher’s transformation. We performed a sensitivity analysis by repeating the same analyses after excluding one study that only reported results for longitudinal changes in BMI and WHtR, but not their baseline values.

## 3. Results

### 3.1. Characteristics of the Studies

The main characteristics and results of the selected studies are shown in Table 1.

We found five original articles assessing the correlation of BMI and WtHr with BF measured by DEXA. Two of the studies were conducted with European participants [25,26]: with children born in Sweden in the study by Karlsson et al., and with Spanish children, or children educated in Spain, in the study by De Miguel Etayo et al. The remaining three studies were conducted in the US [27,28,29]: Barreira et al. recruited local participants in Louisiana, whereas Brambilla et al. and Tuan et al. used the data from children enrolled in the US National Health and Nutrition Surveys (US-NHANES).

The QUADAS tool used to assess the quality of the studies showed that the most remarkable limitation within the studies was the use of not representative samples, which may limit the generalizability of their results. In addition, none of the studies clearly reported whether the authors had made a blind interpretation of the results.

All the articles included in this systematic review were published between 2013 and 2015. The sample sizes ranged from 83 to 5355, and the age of participants ranged from 4.9 to 19 years old.

Most of the studies were cross sectional, only one was a longitudinal study and calculated the correlation between changes in several anthropometric measures in a subsample of children enrolled in the EVASYON study [26], a non-controlled intervention trial for weight loss in overweight and obese adolescents [30]. Since that study was restricted to overweight and obese participants, higher mean BF values were expected compared to other studies.

The BF was reported in kg or as %BF. One of the studies reported the *z*-score of the %BF [29] and another one, the fat mass index (FMI), which is the ratio of the fat mass (kg) and the height squared (m^2^) [26]. Even though all the studies assessed both BMI and WtHr, the correlation was calculated with *z*-score in one study [29], and with changes in the indices (instead of only one-time point values) in another study [26].

Four out of the five studies [13,27,28,29] calculated the *R*^2^ to assess the variability of BF determined by DEXA that could be explained by the variability in anthropometric measures (BMI and WtHr). Other commonly used statistical analyses were correlation coefficients [25,26,29] and beta regression coefficients [26,27,29].

Significant correlations were found between BF measured by DEXA and both BMI and WtHr. In boys, the *R*^2^ ranged from 0.62 to 0.77 for the BMI and from 0.50 to 0.73 for the WtHr [26,29]. In girls, the *R*^2^ ranged from 0.67 to 0.90 for BMI and from 0.49 to 0.70 for the WtHr [26,29]. When boys and girls were studied together, the *R*^2^ ranged from 0.32 to 0.91 for the BMI and from 0.55 to 0.69 for the WtHr [25,27,28].

Only one study [29] calculated the area under the curve (AUC) of BMI and WtHr for the diagnosis of obesity, and the kappa index between those anthropometric measures and DEXA. In that study, the authors had defined obesity as %BF (measured by DEXA) above the sex-age-specific 75th percentile of the reference population (26%–33% in boys and 36%–38% in girls) [31]. In boys, the reported AUC was 0.91 for the BMI and 0.97 for the WtHr, which shows a high capacity of both anthropometric measures to discriminate between obese and non-obese children. Similar results were reported in girls, with an AUC of 0.90 for the BMI and 0.94 for the WtHr. Along with these results, both BMI and WtHr showed high agreement indexes, expressed as weighted kappa for the agreement between quartiles [29].

The main adjusting factor was the age of participants. Only one of the studies adjusted for Tanner stage of pubertal development [26]. Two of the studies stratified their results by sex [26,29], and three out of the five considered ethnicity-race [27,28,29]. One of the studies found a significant interaction with race for the WtHr, but not for the BMI [27]. Another study reported small racial-ethnic variations [29], whereas a third study observed no improvement in their model by adding ethnic group as an additional predictor [28].

Small sample sizes [25,26,27], and the fact that they were not representative were the most important sources of potential bias identified within the studies [25,26,27,28,29]. On the other hand, the fact that one of the studies used FMI to express BF [26] may have increased the risk of bias across studies.

### 3.2. Quantitative Meta-Analysis

**Figure 3:**
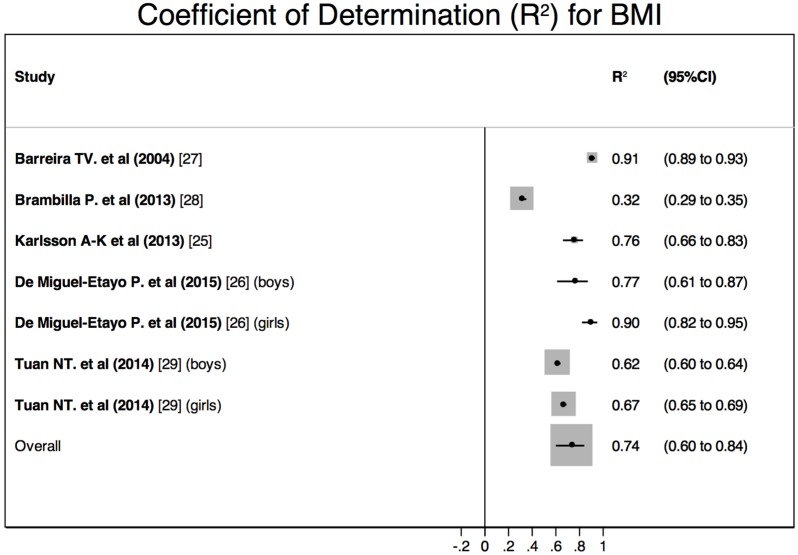
Results of the quantitative meta-analysis: Pooled coefficient of determination (*R*^2^) for BMI to assess BF measured by DEXA.

**Figure 4:**
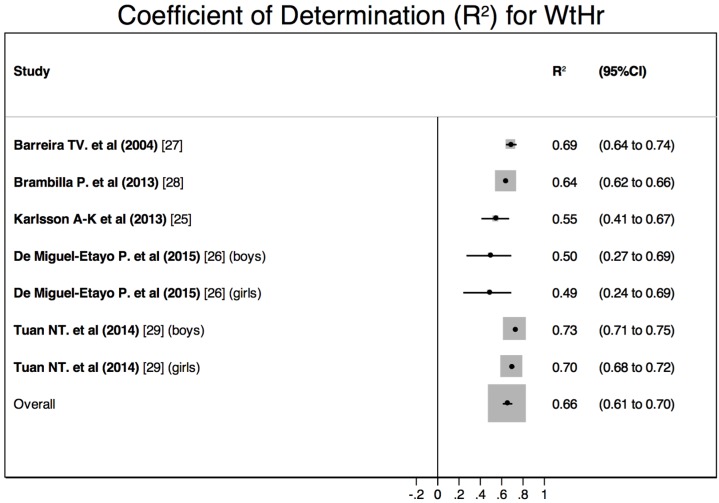
Results of the quantitative meta-analysis: pooled coefficient of determination (*R*^2^) for WtHr to assess BF measured by DEXA.

The quantitative meta-analysis showed a significant between-studies heterogeneity (Figure 3 and Figure 4). The calculated pooled *R*^2^ were 0.74 (95%CI 0.60–0.84) for BMI and 0.66 (95%CI 0.61–0.70) for WtHr. The comparison of these pooled estimates showed no significant differences (*z* = 1.19; *p* = 0.23).

## 4. Discussion

This systematic review showed that both BMI and WtHr were able to explain a high percentage of the variability in BF as measured by DEXA in children, which suggests that they can offer a reasonably good clinical proxy to general obesity. Along with that, we agree with a recent systematic review [12] that concluded that, because BMI and WtHr are easy to obtain, harmless, and affordable, they are good techniques for clinical practice and epidemiological research.

Skinfold measurement has been suggested to be the best anthropometric measure for BF [33,34]. Nevertheless, since weighing and measuring height and body circumferences are easier than measuring skinfolds, other anthropometric measures have been preferred in clinical practice and epidemiological research.

Because body composition changes with age, sex, and ethnicity, the assessment of obesity in children requires sex and age-specific reference tables [35]. Of all anthropometric measurements, BMI is the most commonly used to define obesity. In pediatric populations, an important limitation that affects the comparability among studies is the use of different tables of reference and the lack of a universally accepted cut-off point. Two different systematic reviews found that BMI had high specificity but moderate sensitivity for the diagnosis of obesity during childhood [36,37]. Furthermore, the ability of BMI to identify obesity seems to be modified by nutritional status [38,39].

On the other hand, WtHr is increasingly used because it correlates well not only with BF, but also with visceral abdominal fat and other metabolic risk factors in children [20,40]. The message “keep your waist circumference to less than half your height” applies to all ages [41,42]. A universally accepted single cut-off point would represent an important advantage of the WtHr over other anthropometric measures [43,44]. However, further research is needed in order to assess whether WtHr remains stable during growth [45,46].

Two out of five of the studies included in this systematic review [28,29] concluded that WtHr is better than BMI in predicting total adiposity in children. Those studies were conducted with data from the US-NHANES in 2003–2004 and 2001–2004 respectively, and thus they had an overlap of data that could explain their agreement. Both studies had several strengths, such as large sample sizes (*N* = 2239 and *N* = 5355 respectively), wide age ranges of participants (8–18 and 8–19 respectively), and multi-ethnicity. Brambilla et al. (2013) reported a multivariable adjusted model including age, sex, and race [28], and Tuan et al. (2014) stratified the results by sex and ethnicity-race [29]. Nevertheless, their results may be limited due to a suboptimal adjustment for potential confounders (Tanner stage) and a lack of tests for interaction.

On the other hand, Barreira et al. (2014) [27] found that BMI explained a greater percentage of the BF than the WtHr in a similar multiracial and wide age-range sample. No significant interactions were observed. Similar results were reported by Karlsson et al. [25] and by De Miguel-Etayo et al. [26]. As to the later, it must be considered whether the reported correlation for BMI increased due to the use of FMI to express BF, since, like BMI, it is calculated with height squared (m^2^) in the denominator [26,28].

The quantitative meta-analysis of those studies showed that both BMI and WtHr explained a large percentage of BF measured by DEXA. Although the pooled estimate calculated for the BMI was slightly larger, no significant differences were found.

To test our results, we considered it of interest to repeat the analysis after the exclusion of the study by De Miguel-Etayo et al. [26] because (1) it reported the correlation between the change in the indices and (2) it reported BF as FMI. The correlation between two indices can be fairly assessed by focusing on the correlation between their respective changes in time. Furthermore, changes in time need the use of repeated measurements of each index, and thus they use more information. The sensitivity analysis after the exclusion of that study showed a significantly greater pooled estimate for WtHr (*R*^2^ = 0.72) compared to BMI (*R*^2^ = 0.65) (*z* = −6.82. *p* < 0.01). De Miguel-Etayo et al. reported very high correlations for BMI and very low correlations for WtHr. As discussed above, the use of FMI to express BF could be related to these findings. Besides, since BMI shows stronger correlations with BF in overweight and obese children [17], all participants being overweight or obese adolescents may have influenced their findings. Whether the nutritional status affects the accuracy of the WtHr for the classification of obesity needs further research. 

It is important to highlight that the correlation coefficient shows relation but not agreement. The significant *p* values reported in the studies included in this review only reinforce that two methods designed to measure the same variable are related [12,47]. In order to synthesize the results, the confidence intervals would have been more informative. On the other hand, the assessment of the agreement between two measuring techniques represents an important statistical tool, since it helps identify the optimal cut-off points to maximize sensitivity and specificity. Agreement can be tested by the intraclass correlation coefficient and the Bland and Altman method for quantitative variables, and by the Kappa statistics and the AUC for qualitative variables. Only one of the studies included in this review assessed the agreement between DEXA and anthropometric measures (BMI and WtHr) [29]. Taking the standard reference for obesity as a %BF above the 75th age and sex-specific percentile [31], authors found higher agreement and higher AUC for the *z*-score of the WtHr.

This systematic review contributes to previous literature because we calculated a pooled estimate for the correlation between both BMI and WtHr with BF measured by DEXA, and we compared these pooled estimates obtaining no significant differences. Nevertheless, some limitations may be pointed out in this review. The potential bias within and across the studies has already been discussed. Additionally, even though we conducted our research in two important data bases (PubMed/Medline and Embase), the possibility of a selection bias must also be taken into account. Finally, in this review only a total of five studies were finally included, which may suggest we established very narrow inclusion criteria, but there may also be a risk of publication bias.

## 5. Conclusions

We found that both BMI and WtHr were strongly correlated with BF measured by DEXA; thus, both can be used to diagnose obesity in pediatric populations when more sophisticated techniques are not available. The reviewed literature did not provide a conclusive answer about whether WtHr is superior to BMI to assess general obesity, since no significant differences were found when we compared the pooled *R*^2^.

## Figures and Tables

**Table 1:** Characteristics of the five studies that assessed the correlation of both body mass index (BMI) and waist-to-height ratio (WtHr) with body fat (BF) measured by dual-energy X-ray absorptiometry (DEXA).

Authors (Year)	Design	Simple Characteristics	Age (Years)	Mean %BF (SD)	Fat Mass Assessment	BMI or WtHr	Results *			Other Findings
							*R*^2^ (95%CI)	Beta Coefficient	AUC	
Barreira, T.V., et al. (2014) [27]	Cross-sectional	*N* = 382; 180 boys (84 African Americans (AA) & 96 Whites) and 202 girls (118 AA & 84 Whites).	5–18	Boys: Whites: 24.0 (9.1) AA: 24.0 (10.0) Girls: Whites: 31.1 (8.4) AA: 31.8 (9.6)	Total fat mass (kg)	BMI	0.91 (0.89–0.93)	Significant linear relationship		No significant interaction with race or sex.
						WtHr	0.69 (0.64–0.74)	Significant linear relationship		Significant interaction race and sex.
Brambilla, P., et al. (2013) [28]	Cross-sectional (US-NHANES 2003–2004)	*N*= 2339; 1221 boys (326 Whites, 453 Blacks, 373 Mexicans, 69 other races) & 1118 girls (321 Whites, 387 Blacks, 348 Mexicans, 62 other races).	8–18	Whites: 29.0 (7.7) Blacks: 26.0 (8.3) Mexicans: 29.8 (7.7) Others: 28.3 (7.8)	%BF	BMI	0.32 (0.29–0.35) (unadjusted) 0.68 (age & sex) 0.70 (age, sex & race)			Found no practical advantage to add the ethnic group as further predictor in the model.
						WtHr	0.64 (0.62–0.66) (unadjusted) 0.80 (age & sex) 0.80 (age, sex & race)			
Karlsson, A.-K., et al. (2013) [25]	Cross-sectional	*N* = 100; 55 boys and 45 girls, moderately preterm at birth.	4.9–5.2	Boys: 17 (6) Girls: 21 (6)	Total fat mass (kg)	BMI	0.76 (0.66–0.83)			
						WtHr	0.55 (0.41–0.67)			
De Miguel-Etayo, P., et al. (2015) ϕ [26]	Longitudinal (EVASYON treatment program) [30]	*N* = 83; 43 boys and 40 girls, overweight or obese [32]	13–16	Boys: 33.3 (31.5–35.1) Girls: 39.9 (37.9–41.9)	Changes in FMI.	Changes in BMI	Boys: 0.77 (0.61–0.87) Girls: 0.90 (0.82–0.95)	Boys: 0.68, Girls: 0.66 (unadjusted) Boys: 0.68, Girls: 0.66 (age) Boys: 0.68, Girls: 0.65 (age and Tanner)		
						Changes in WtHr	Boys: 0.50 (0.27–0.70) Girls: 0.49 (0.24–0.69)	Boys: 33.91, Girls: 19.04 (crude model) Boys: 33.57, Girls: 18.37 (age) Boys: 33.61, Girls: 18.53 (age and Tanner)		
Tuan, N.T., et al. (2014) [29]	Cross-sectional (US-NHANES 2001–2004)	*N*= 5355; 2792 boys (796 Whites, 962 Blacks, 818 Mexicans, 216 other races) & 2563 girls (742 Whites, 809 Blacks, 809 Mexicans, 203 other races).	8–19	Boys: Whites: 25.7 (0.5) Blacks: 22.8 (0.2) Mexican: 27.3 (0.3) Girls: Whites: 32.5 (0.4) Blacks: 31.5 (0.3) Mexican: 33.7 (0.3)	*z*-score of the %BF Obesity was defined as %BF by DEXA > 75th age & sex specific percentile (26%–33% in boys & 36%–38% in girls) [31].	*z*-score of the BMI	Boys: 0.62 (0.60–0.64) Girls: 0.67 (0.65-0.69)	Boys: 0.79 Girls: 0.82 (*z*-BMI) (all races)	Boys: 0.91 Girls: 0.90 (all races)	Kappa ^+^ Boys: 0.59 Girls: 0.60 (all races) Sex and ethnicity-race variations.
						*z*-score of the WtHr	Boys: 0.73 (0.71–0.75) Girls: 0.70 (0.68–0.72)	Boys: 0.86 Girls: 0.84 (*z*-WtHr) (all races)	Boys: 0.97 Girls: 0.94 (all races)	Kappa ^+^ Boys: 0.71 Girls: 0.64 (all races) Sex and ethnicity-race variations.

* Adjusting factors appear in brackets; ^+^ Weighted Kappa for the agreement between quartiles; ϕ This study reported Pseudo-*R*^2^ instead of *R*^2^; and %BF was reported as median (interquartile range); SD: standard deviation; SE: standard error; BMI: body mass index; WtHr: waist-to-height ratio; *R*^2^: coefficient of determination; AUC: Area under the curve; DEXA: dual-energy X-ray absorptiometry; %BF: body fat percentage; FMI: Fat mass index; NHANES: National Health and Nutrition Examination Survey.

## Abbreviations

The following abbreviations are used in this manuscript:

AUC	area under the curve
BF	body fat
%BF	body fat percentage
BMI	body mass index
DEXA	dual-energy X-ray absorptiometry
FMI	fat mass index
NHANES	national health and nutrition examination survey
*r*	correlation coefficient
*R*^2^	coefficient of determination
WtHr	waist to height ratio
WC	waist circumference
95% CI	95% confidence interval
